# Antenatal care in rural Bangladesh: Gaps in adequate coverage and content

**DOI:** 10.1371/journal.pone.0205149

**Published:** 2018-11-19

**Authors:** Abu Bakkar Siddique, Janet Perkins, Tapas Mazumder, Mohammad Rifat Haider, Goutom Banik, Tazeen Tahsina, Md. Jahurul Islam, Shams El Arifeen, Ahmed Ehsanur Rahman

**Affiliations:** 1 Maternal and Child Health Division, International Centre for Diarrhoeal Disease Research, Bangladesh (icddr,b), Dhaka, Bangladesh; 2 Health Department, Enfants du Monde, Geneva, Switzerland; 3 Department of Health Promotion, Education and Behavior, Norman J Arnold School of Public Health, University of South Carolina, Columbia, South Carolina, United States of America; 4 National Newborn Health Program & Integrated Management of Childhood Illness, Directorate General of Health Services, Ministry of Health and Family Welfare (MOH&FW), Dhaka, Bangladesh; Children's Mercy Hospitals and Clinics Department of Pathology and Laboratory Medicine, UNITED STATES

## Abstract

**Introduction:**

Antenatal care (ANC) has long been considered a critical component of the continuum of care during pregnancy, with the potential to contribute to the survival and thriving of women and newborns. Although ANC utilization has increased in over the past decades, adequate coverage and content of ANC contacts have fallen under increased scrutiny. The objectives of this article are to describe the coverage and content of ANC contacts in the context of rural Bangladesh.

**Methods:**

A community-based, cross-sectional household survey was conducted in two sub-districts of Netrokona district, Bangladesh in 2016. A total of 737 women with a recent birth outcome were interviewed. Respondents reported on the ANC contacts and the content of these contacts. Descriptive statistics were used to report coverage and content of ANC contacts stratified by covariates. Chi-square tests were performed to explore whether the estimates are different among different categories and significant differences were reported at p<0.05.

**Results:**

Around 25% of women attended at least four ANC contacts, with only 11% initiating ANC in the first trimester of pregnancy. Blood pressure was measured in almost all of the ANC contacts (92%), and abdominal examination performed in 80% and weight measured in 85% of ANC contacts. Urine tests were conducted in less than half of the ANC contacts, whereas blood screening tests and ultrasound were conducted in 45% contacts. Health care providers counselled women on danger signs in only 66% of the ANC contacts. Overall, the content of facility-based ANC contacts were better than home-based ANC contacts across all components.

**Conclusions:**

Adequate coverage of ANC remains poor in Netrokona, Bangladesh and important gaps remain in the content of ANC contacts when women attend these services.

## Background

Antenatal care (ANC) has long been considered a critical component of the continuum of care for women during pregnancy, with the potential to contribute to the survival and thriving of women and newborns [1, 2]. This essential service allows women to be screened during their pregnancies for pre-existing conditions and potential complications, allows for initiation of timely and appropriate treatment, and provides a platform for women to receive counselling, which can support them to protect their health and that of their baby throughout the antenatal, birth and postnatal periods [3]. Moreover, ANC is becoming increasingly important as a service as the world undergoes an obstetric transition [4]. In this transition, preventable maternal mortality is becoming predominantly the result of indirect causes and non-communicable diseases, which requires more individualized care [3, 5]. ANC can provide an optimal platform for catering the individual care by screening and timely management [4, 6]. Promisingly, utilization of ANC has been increasing steadily throughout the past decades, with 86% women worldwide now attending at least one ANC contact and 62% receiving at least four ANC contacts between conception and birth [7]. However, even as ANC utilization has increased over the past two decades, the content and quality of this care have fallen under increased scrutiny, as poor quality compromises the potential benefits of care [8]. With the new targets set out in the Sustainable Development Goals (SDGs) aiming to reduce maternal and newborn deaths to unprecedented levels, and the ambitious ‘Survive, Thrive, Transform’ agenda of the Global Strategy for Women’s, Children’s and Adolescent’s Health, ensuring the quality of maternal and newborn health (MNH) services, including ANC, is as important as ever [9, 10].

The World Health Organization (WHO) recently updated its ANC guidelines based on the global evidence base [3]. The new guidelines are notable in their adoption of a human rights-based approach and a focus on people-centred care. This emphasizes not only clinical service provision but also the experience of care; so that adolescent girls and women are able to benefit from a positive pregnancy experience. Moreover, it is now recommended that each woman attend eight of more routine ANC contacts between conception and birth, rather than the four or more suggested by the previous model [3, 11]. The new guidelines are more expansive and comprehensive than the previous model, and clearly have the potential to improve the pregnancy experience and outcomes. During the Millennium Development Goals-era, the global coverage of ANC contacts inched forward, but many countries struggled to ensure adherence to the recommendations contained in the previous model [12]. Based on this experience, it will be challenging for the countries with limited resources to ensure the adherence to the more comprehensive recommendations [12]. A number of studies have explored the degree to which the recommended content of ANC contacts are adhered to in different countries. In general, these studies demonstrate the poor status and existing gaps related to the content of ANC contacts, even in the context of high-resource settings [13, 14], much less in low and middle-income countries (LMICs) [15–17].

Bangladesh has made impressive gains in reducing maternal and neonatal mortality over the past several decades, but total number and rates of these deaths remain too high. Moreover, the latest Bangladesh Maternal Mortality Survey (BMMS-2016) suggests that progress in reducing maternal mortality has stalled [18]. Use of key MNH services remains critically low. Indeed, only 37% pregnant women attend at least four ANC contacts, 47% of births occur in health facilities and 48% (6% in the case of home-based births) of women receive postnatal care from a skilled health-care professional within the first two days after birth [18]. While the BMMS-2016 revealed that use of skilled health services during pregnancy has increased over the past decade, this has not translated into an expected reduction in maternal mortality between 2010 and 2016. This suggests that focusing solely on increasing coverage of these services is not sufficient to translate into improved health. The content and the quality of these contacts must also be ensured [3].

In order to identify where to focus efforts to increase the coverage and improve the WHO recommended content of ANC contacts, so that it can more fully contribute to the health and well-being of women and their newborns, a more complete picture of the current status is required. Few studies to date have examined in depth the adequacy of coverage and the content of ANC in Bangladesh [19, 20], particularly in rural settings where access to quality health services remains the most precarious [18, 21–23]. This is of critical importance as the country re-examines best strategies for reducing excess maternal and newborn mortality after the recent stalling and to achieve the ambitious SDG targets [24]. It is also relevant at the international level as the global community strives to gain a more complete picture of coverage and quality of ANC in different contexts in order to advance global initiatives [25, 26]. In this article, we explore the coverage and the content of ANC contacts in rural Bangladesh.

## Materials and methods

### Study design and settings

A community-based, cross-sectional household survey was conducted in hard-to-reach two sub-districts, Barhatta and Kalmakanda, of Netrokona district in Bangladesh in 2014. Netrokona is located approximately 200 kilometres north of Dhaka, the capital of Bangladesh. It is one of the 14 lowest performing districts of Bangladesh in terms of newborn and child mortality rates [27]. Netrokona’s landscape is dominated by four major rivers and abundant wetlands known as *haors*. Agriculture and fishing are the primary sources of income. The sub-district of Kalmakanda has a land area of 377 square kilometres and a total population of around 272,000. Barhatta covers 220 square kilometres of land with a total population of approximately 180,000.

### Study population, sample size and sampling

Married women with a recent history of birth (within the 12-month period preceding the survey) who were permanent residents of the selected sub-districts were eligible to be included in the study. We adopted a multistage cluster sampling to identify eligible participants for the household survey. In the first stage, four unions (the smallest administrative unit of Bangladesh with an average population of 30,000) were randomly selected from each sub-district. In the second stage, forty clusters (average population of approximately 1,000) were selected from the selected unions using probability proportional to size (PPS) sampling technique. All eligible respondents were included from the selected clusters. A total of 737 women with a recent history of birth were interviewed with a non-response rate of less than 5%.

### Data collection

The household survey was conducted in September-October 2016. At first, a sketch map was drawn for each of the selected clusters representing boundaries, landmarks and bari (extended household) locations. All households in the selected clusters were enumerated and listed. Then all women with a recent history of birth were screened and identified from the listed households. In the second stage, interviewer-administered structured questionnaires were used for interviewing all eligible women. The questions were adapted from the tools used in Bangladesh Demographic and Health Survey (BDHS), Bangladesh Maternal Mortality Survey (BMMS), Multiple Indicator Cluster Survey (MICS) and other relevant studies [28–31]. The questionnaire started with questions regarding personal and socioeconomic information such as age, education level, marital status and employment status followed by questions related to utilization of routine and emergency obstetric care, the coverage and content of ANC contacts attended and knowledge related to pregnancy and birth.

For quality assurance, the questionnaire was pre-tested in non-selected clusters of the selected unions. Interviewers were locally recruited to facilitate the data collection processes, as they would be familiar with the local context, culture and dialects. Experienced facilitators, trainers and field supervisors trained the data collectors.

### Data analysis

#### Background characteristics

Age and education level of the women and their husbands were transformed into categorical variables. Due to small numbers, all other religions except ‘Muslims’ were grouped into one category and coded as “others”. We used the standard steps of principal component analysis to generate the socio-economic indices of the households that were interviewed, based on which the wealth quintile was generated [32, 33]. Household-level variables such as household possessions; floor construction materials, wall, and roof; drinking water source; toilet facilities; and ownership of land and domestic animals were used to generate this index.

#### Coverage of ANC contacts

The coverage of ANC contacts was considered as the primary outcomes of interest. ANC was operationally defined as any medical contact with a formal health care provider during pregnancy for routine check-ups. Facility-based service providers such as doctors, midwives, nurses, family welfare visitors (health service provider with three years of in-service training in reproductive and maternal health services), community health care providers (health service providers responsible for community clinics), and community-based health workers (such as health assistants, family welfare assistants, community-based skilled birth attendants, and NGO health workers) were considered as formal health care providers. The following indicators were considered pertaining to coverage of ANC contacts:

- Any ANC: attended at least one ANC contact from a formal health care provider during pregnancy
- Early initiation of ANC: attended the first ANC contact within the first 12 weeks of pregnancy as recommended by WHO [3]
- ANC in all trimesters: attended at least one ANC contact in each trimester of pregnancy as recommended by WHO [3]
- ≥4 ANC: attended four or more ANC contacts during pregnancy as recommended by the Government of Bangladesh [24]
- Adequate coverage of ANC: ‘early initiation of ANC’ *and* ‘ANC in all trimesters’ and ‘≥4 ANC’ during the pregnancy period.

At first, we used descriptive statistics (proportions) to report the indicators selected for representing the coverage of ANC contacts. The estimates were also presented stratified by the following background characteristics: age of the women and husbands, education status of the women and husbands, religion and wealth quintile. Then we separately presented the associations between coverage of ANC contacts and background characteristics through chi-square tests.

Content of ANC contacts: The content of ANC contacts was considered as the secondary outcome of interest. The following indicators were selected in relation to the content of ANC contacts based the recent WHO recommendations and Bangladesh Maternal Health Strategy and SoPs [3, 24] and the availability of data from the survey:

- Physical examinations: measurement of blood pressure, examination of the abdomen, measurement of weight;
- Screening tests: blood test, urine test, ultrasonography;
- Counselling topics: counselling on maternal and newborn danger signs, counselling on the importance of seeking care from appropriate places and health care providers for routine and emergency obstetric services

Initially, the content of ANC contacts was analyzed considering individual women as the unit of analysis. Specific content of ANC contacts were reported using descriptive statistics stratified by frequency (content received at least once, content received more than one,) and timing (content received during the first ANC contact, content received in the first, second and third trimesters and content received in all trimesters).

The following composite indicators to assess content of ANC contacts were generated from the individual content of ANC contacts:

- Received all three physical examination components
- Received all three screening test components
- Received counselling on both counselling topics
- Received all eight components of ANC contacts related to physical examination, screening tests and counselling.

Descriptive statistics were used to present the composite indicators stratified by background characteristics (described in analysis plan for coverage of ANC contacts). Chi-square tests were used to report whether there is any difference in content of ANC contacts by categories of individual-level covariates.

Secondly, the content of ANC contacts was analyzed considering each contact as the unit of analysis. Maintenance of privacy was added as an additional content of ANC contacts during this analysis. The estimates were stratified by place of attending the service and type of health care provider. Husbands accompanying their wives during the ANC contact was also considered as a stratifying variable. The relationships between the individual content of care and different covariates were explored with proportion tests (z test).

Data were analysed using Stata 14.0 (StataCorp. 2015. Stata Statistical Software: Release 14. College Station, TX: Stata Corp LP). All estimates were generated after necessary weighting since we have adopted a multi-stage cluster sampling. Any significant relationship was reported at p<0.05.

### Ethical approval and consent to participate

Ethical approval to conduct the study was obtained from the Institutional Review Board of icddr,b (Protocol Number: PR 14024). The Institutional Review Board of icddr,b consists of two independent committees, i.e. Research Review Committee and Ethics Review Committee. Administrative approval was obtained from the local MOHFW health managers before the commencement of survey.

All study participants were fully informed that their participation was voluntary and that they had the right to withdraw from the study at any time. They were also informed that refusal to participate in the study would not involve any penalty. Written and informed consent was obtained from each participant once they were fully informed. Privacy, anonymity and confidentiality of the participants were strictly maintained during data collection and analysis.

## Findings

Table 1 presents the background characteristics of the study population. The mean age of women was 26 years, with half of the respondents between the age of 25–34. Nearly half of the women had less than five years of formal education, with 44% having completed 5–9 years and only 8% having completed 10 or more years of formal schooling. Approximately 58% of their husband less than five years of formal education, with one third having completed 5–9 years and only 9% having completed 10 or more years or formal schooling. The majority of respondents were Muslim (88%).

**Table 1:** Background characteristics of women with a recent history of birth (N = 737).

	%	n
**Age of woman**		
15–24	40.3	297
25–34	50.1	369
35+	9.5	70
Mean age in years (SD)	26.4 (5.6)	737
**Education of women** **(years of schooling)**		
0–4 years	47.9	353
5–9 years	43.7	322
≥10 years	8.3	61
Mean years of schooling (SD)	4.6 (3.3)	737
**Education of husband** **(years of schooling)**		
0–4 years	58.3	430
5–9 years	31.6	233
≥10 years	9.3	69
Mean years of schooling (SD)	4.1 (3.6)	737
**Religion**		
Other	11.5	85
Muslim	88.3	651
**Wealth Quintile**		
Lowest	19.9	147
Second	20.3	149
Middle	20.0	148
Fourth	19.9	146
Highest	19.9	147

Table 2 presents the coverage of ANC contacts among women who had a recent history of birth. Less than two-thirds of the women attended any ANC with a formal health care provider during their most recent pregnancy. Only 11% of women had early initiation of ANC. Less than one-tenth (9%) of them attended ANC in all trimesters. Around one-quarter attended the nationally recommended ≥4 ANC. Only 7% had adequate coverage of ANC contacts (‘ANC in all trimesters’ *and* ‘≥4 ANC’). Only 1 women had attended the WHO recommended 8 or more ANC contacts during her most recent pregnancy. Higher education of women and their husbands were associated with attending any ANC (p = 0.00), early initiation of ANC (p = 0.00), ANC in all 3 trimesters (p = 0.00), 4 or more ANC (p = 0.00) and adequate coverage of ANC contacts (‘ANC in all trimesters’ *and* ‘≥4 ANC’) (p = 0.00). Religion was also associated with early initiation of ANC (p = 0.00), ANC in all 3 trimester (p = 0.00) as the practices were better among non-Muslims. Significant associations were also observed between wealth and attending 4 or more ANC and adequate coverage of ANC contacts (‘ANC in all trimesters’ *and* ‘≥4 ANC’) (p = 0.00). The adjusted associations (multiple logistic regression) are presented in S1 Table.

**Table 2:** Coverage of ANC contacts; percent distribution among women with a recent history of childbirth by background characteristics (N = 737).

	Any ANC	P value	Early initiation of ANC	P value	ANC in all 3 trimesters	P value	≥4 ANC	P value	Early initiation of ANC + ≥4 ANC	P value	ANC in all 3 trimesters + ≥4 ANC	P value
	%		%		%		%		%		%	
**Age of woman**												
15–24	69.2	0.05	12.2	0.52	9.3	0.61	27.8	0.23	7.8	0.37	7.5	0.40
25–34	61.7		11.8		8.8		22.3		7.4		7.4	
35+	58.3		6.3		4.8		26.5		3.2		3.2	
**Education- women**												
0–4 years	59.4	0.00	10.9	0.00	6.8	0.00	21.1	0.00	4.9	0.00	4.9	0.00
5–9 years	64.4		8.5		6.9		22.1		5.9		5.6	
≥10 years	93.6		30.0		28.6		61.7		26.8		26.8	
**Education-husband**												
0–4 years	59.6	0.00	9.1	0.002	6.5	0.00	21.0	0.00	4.5	0.00	4.5	0.00
5–9 years	67.9		10.9		7.8		24.6		6.4		6.4	
≥10 years	84.2		27.0		23.8		51.9		25.4		23.8	
**Religion**												
Other	70.5	0.16	28.7	0.00	25.1	0.00	38.2	0.00	21.2	0.00	21.2	0.00
Muslim	63.6		9.2		6.5		23.2		5.4		5.2	
**Wealth Quintile**												
Lowest	53.9	0.00	6.0	0.23	4.5	0.12	24.5	0.00	4.5	0.00	4.5	0.01
Second	66.3		9.4		5.9		20.8		4.4		4.4	
Middle	56.9		10.5		9.2		19.1		5.6		5.6	
Fourth	63.2		13.4		8.1		22.5		6.6		6.6	
Highest	81.9		17.7		15.5		38.5		14.7		14.0	
**Total**	64.5		11.4		8.6		25.0		7.2		7.0	

**Fig 1:**
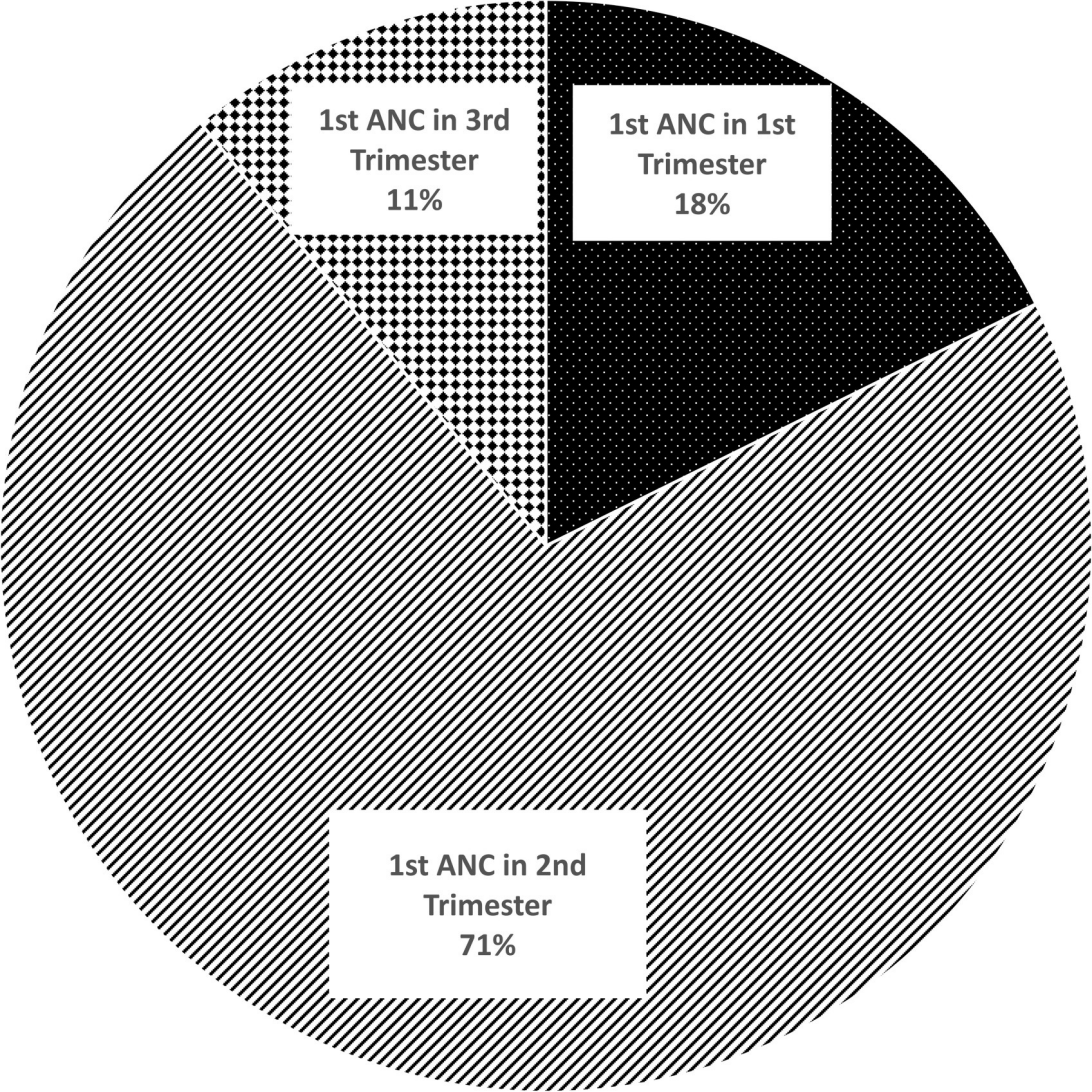
Timing of the first ANC contact, percentage distribution among women who attended ANC (N = 454).

Fig 1 presents the timing of initiation of ANC among women who had attended at least one ANC contact with a formal health care provider during their most recent pregnancies. Among these women, less than one-fifth (18%) attended their first ANC contact within the first 12 weeks (1^st^ trimester) of pregnancy and the majority (71%) of them attended the first ANC contact in the second trimester. Around 11% of women waited until the final trimester to attend their first ANC contact. Out of those who initiated ANC early, 76% attended ANC in all trimesters. Moreover, the odds of receiving 4≥ ANC was eight times higher (CI 4.1 16.3) among those who attended ANC in the 1^st^ trimester compared to those who did not attend in the 1^st^ trimester.

Table 3 presents the percent distribution of women receiving different content of ANC during their ANC contacts. Among women who attended any ANC during their most recent pregnancy, over 90% had their blood pressured measured, weight measured and an ultrasound conducted at least once during the pregnancy. Around four-fifths of women had abdomen examination performed (84%)and urine tested (78%) at least once during pregnancy. Fewer than two-thirds (71%) were counselled on maternal and newborn danger signs at least once and around four-fifths (82%) were counselled on the care seeking practices at least once during pregnancy. Only around one-quarter had their blood and urine tested and an ultrasound conducted more than once. Only around half of respondents were counselled on danger signs (56%) and care seeking practices (62%) more than once during their ANC contacts. Only 16% women had their blood pressure measured, abdomen examined and weight measured during the first trimester. However, more than two-thirds of women who had ANC had their blood pressure measured, abdomen examined, weighed measured and urine tested in the first and the third trimesters of pregnancy. Only around one-tenth (11%) of respondents had an ultrasound conducted in the first trimester. Around half of them had an ultrasound conducted in the third trimester.

**Table 3:** Status of receiving content of ANC contacts, percent distribution among women who have attended ANC by frequency and timing of ANC contacts (N = 454).

	Physical examination			Screening tests			Counselling	
	Blood pressure	Abdominal examination	Weight measured	Urine test	Blood test	USG	DSC	CSC
	%	%	%	%	%	%	%	%
**Frequency**								
At last one	95.1	83.6	90.0	78.1	57.9	99.8	70.7	81.5
More than once	75.9	65.5	67.9	27.8	22.0	23.0	55.7	62.4
**Timing**								
1^st^ visit	94.4	79.2	87.1	72.7	46.1	38.0	68.4	77.6
1^st^ trimester	16.1	16.2	16.1	15.8	12.6	10.8	13.7	16.0
2^nd^ trimester	84.3	74.3	79.5	68.4	50.1	43.7	63.7	73.2
3^rd^ trimester	76.1	68.9	73.3	63.1	50.6	42.8	57.6	66.7
All 3 trimesters	12.5	12.3	12.7	12.5	10.7	9.7	10.5	12.2

Table 4 presents the relationship between background characteristics and status of women receiving different content of ANC during their most recent pregnancy. Out of those who attended any ANC, around three-quarters (77%) received all three components of physical examination. Nearly half had the three screening tests conducted (52%) and were counselled on both topics considered (46%). Only one-third received all eight components of ANC during their most recent pregnancy. Wealth (p = 0.000), religion (non-Muslim) (p = 0.000) and women’s age (p = 0.020), women’s education (p-0.001) (were associated with receiving all eight components of ANC. The adjusted associations (multiple logistic regression) are presented in S2 Table

**Table 4:** Relationships between status of receiving content of ANC and background characteristics, among women who have attended ANC (N = 454).

Background characteristic	Physical examination (blood pressure& abdominal examination& weight measured)		Screening tests (urine test& blood test& USG)		Counselling (DSC & CSC & privacy)		All	
	%	P value	%	P value	%	P value	%	P value
**Age of woman**								
15–24	74.1	0.048	42.9	0.000	43.6	0.586	29.1	0.020
25–34	81.1		61.2		47.4		43.0	
35+	65.3		43.0		39.5		38.8	
**Education- women**								
0–4 years	74.8	0.213	42.5	0.000	42.4	0.076	30.5	0.001
5–9 years	75.9		54.2		43.4		36.1	
≥10 years	86.8		76.2		61.5		61.0	
**Education-husband**								
0–4 years	70.8	0.004	43.9	0.000	40.0	0.009	30.3	0.000
5–9 years	80.8		54.7		47.0		36.3	
≥10 years	90.8		76.6		64.0		64.9	
**Religion**								
Other	84.3	0.137	74.2	0.000	54.2	0.120	58.3	0.000
Muslim	75.6		48.4		43.8		33.5	
**Wealth Quintile**								
Lowest	79.3	0.004	32.2	0.000	36.7	0.000	23.4	0.000
Second	67.9		44.5		29.6		23.1	
Middle	70.3		51.9		48.8		37.0	
Fourth	74.5		53.0		49.6		38.3	
Highest	88.7		68.8		57.8		54.6	
**Total**	76.8	-	51.6	-	45.2	-	36.6	-

Table 5 presents the place where women attended ANC contacts and type of health care provider providing the service. There were a total of 1249 ANC contacts attended by 454 women during the pregnancy period. Out of these ANC contacts (N = 1249), around 14% were carried out at homes, 65% in public hospitals and facilities, and 21% in NGO and private health facilities. Nearly half of the ANC contacts were with medical doctors and around one-third with community-based health workers. Husbands accompanied their wives during nearly two-thirds of these ANC contacts.

**Table 5:** Place and type of provider, percent distribution among ANC contacts (N = 1,249).

Background characteristics	%	N
**Place**		
Home	14.4	180
Public facility	64.9	811
Private and NGO facility	20.7	258
**Provider**		
Doctors	46.5	581
Nurses, Midwives, Paramedics (SACMO and FWV)	18.1	226
Community Health Workers (CSBA, HA, FWA and CHPC)	35.4	442
**Trimester**		
1^st^ and 2^nd^	59.2	739
3^rd^	40.8	510
**Husband accompanied**		
Yes	60.3	753
No	39.7	497

Table 6 presents the status of receiving different components of ANC and the relationship with place and type of health care provider for ANC contacts. Among all ANC contacts, blood pressure was measured universally (92%). Abdominal examination was performed and weight was measured in four-fifths of all ANC contacts. Regarding screening tests, urine testing was conducted in fewer than half of ANC contacts, whereas blood testing and ultrasound were conducted in one-third of the contacts. Health care providers counselled women on danger signs in nearly two-thirds of the ANC contacts and counselled women on care seeking practices for routine and emergency services during three-quarters of the contacts. Privacy was reported to be maintained in two-thirds of the ANC contacts.

**Table 6:** Status of receiving different contents of ANC contacts and their relationship with place and type providers, percent distribution among ANC contacts (N = 1249).

Characteristics	Blood pressure	p	Abdominal examination	p	Weight measured	p	Urine test	p	Blood test	p	USG	p	DSC	p	CSC	p	Privacy	p
	%		%		%		%		%		%		%		%		%	
**Place**																		
Home	84.6	0.000	94.6	0.000	75.8	0.000	26.2	0.000	12.9	0.000	7.0	0.000	53.3	0.000	68.9	0.049	62.1	0.309
Facility	93.5		77.9		86.6		47.6		35.5		36.7		67.7		75.8		66.0	
Public	93.2	0.393	75.1	0.000	83.8	0.000	46.7	0.300	34.9	0.447	34.1	0.002	64.9	0.001	74.3	0.043	70.9	0.000
Private & NGO	94.7		86.6		95.4		50.4		37.5		44.8		76.6		80.5		50.5	
**Provider-cat 1**																		
Doctors	92.4	0.843	66.3	0.000	80.9	0.000	60.2	0.000	47.9	0.000	61.9	0.000	53.8	0.000	66.8	0.000	76.8	0.000
Others	92.1		92.5		88.7		31.0		18.7		6.8		75.9		81.8		55.5	
**Provider-cat 2**																		
doctors, nurses and paramedics	92.5	0.658	73.7	0.000	84.2	0.236	52.4	0.000	37.7	0.000	45.9	0.000	61.4	0.000	71.6	0.000	67.5	0.033
Community health workers	91.8		92.2		86.7		30.3		22.4		7.8		73.4		80.7		61.5	
**Trimester**																		
1^st^ and 2^nd^	91.4	0.364	81.0	0.458	85.5	0.626	51.0	0.000	33.2	0.393	28.8	0.001	66.9	0.242	75.5	0.496	66.1	0.535
3^rd^	91.4		79.3		84.5		35.1		30.9		37.7		63.7		73.8		64.4	
**Husband present**																		
Yes	93.4	0.061	75.3	0.000	85.2	0.884	55.1	0.000	42.0	0.000	46.0	0.000	66.4	0.467	76.5	0.087	66.5	0.309
No	90.5		87.9		84.9		28.5		17.6		11.8		64.4		72.2		63.7	
**Total**	92.2		80.3		85.1		44.5		32.3		32.4		65.6		74.8		65.4	

Facility-based ANC contacts had more complete content of care than home-based ANC contacts, with the exception of abdominal examination, which was significantly higher in home-based ANC contacts (95% vs 78%, p = 0.000). Blood pressure was measured in 94% of facility-based ANC contacts, in contrast to 85% of home-based ANC contacts (p = 0.000). Similarly, weight was measured more frequently in facility-based ANC contacts (87%), compared to home-based ANC contacts (76%), (p = 0.000). Urine testing was performed in nearly half of the facility-based ANC contacts, whereas it was performed in only one-quarter of the home-based ANC contacts (p = 0.000). Blood testing was conducted in one-third of facility-based ANC contacts, and was only performed in 13% in home-based ANC contacts (p = 0.000). Women were counselled on danger signs in two-thirds of facility-based ANC contacts, compared to half of the home-based ANC contacts (p = 0.000). There was no notable difference between home-based and facility-based contacts regarding maintenance of privacy during ANC contacts.

ANC content was somewhat better in private facility-based contacts compared to public facility-based contacts, as abdominal examination (p = 0.000), weight measurement (p = 0.000), ultrasound (p = 0.002) and danger sign counselling (p = 0.001) were more frequently performed. However, privacy was more often maintained in public facility-based ANC contacts (71%) than private facility-based ANC contacts.

The frequency of conducting screening tests was relatively higher among ANC contacts with doctors. Urine testing was performed in 60% ANC contacts with doctors, compared to only 31% of ANC contacts with other providers (p = 0.000). Similarly, an ultrasound was conducted in 62% of ANC contacts with doctors compared to only 7% among ANC contacts with other providers (p = 0.000). Privacy was more often maintained in ANC contacts with doctors (77% vs 56%, p = 0.000). Contrary to this trend, more women were counselled on danger signs and appropriate care seeking practice during ANC contacts with other providers (danger sign counselling 76%; care-seeking counselling 82%) compared to ANC contacts with doctors (danger sign counselling 54%, care-seeking counselling 67%) (p = 0.000). Regarding physical examinations, they were reasonably high in ANC contacts with doctors and others with no notable difference, with the exception of abdominal examination which was substantially higher in ANC contacts with other providers (93% vs 63%, p = 0.000).

Ultrasound was performed more frequently in the third trimester ANC contacts (38% vs 29%, p = 0.001); whereas u testing was conducted more frequently in the first and second trimester ANC contacts (51% vs 35%, p = 0.000). Urine testing (55% vs 29%, p = 0.000), blood testing (42% vs 18%, p = 0.000) and ultrasound (46% vs 12%, p = 0.000) were performed more frequently during the ANC contacts in which the husband was present.

## Discussion

When conducted appropriately and completely, ANC has the potential to save lives and make a major contribution to Bangladesh’s advancement towards achieving the SDGs and establishing the world envisioned within the Global Strategy for Women’s, Children’s and Adolescents’ Health. The findings of this study reveal that the coverage of this essential service remains critically low in Netrokona, an underperforming district in Bangladesh. The findings also highlight that even when women attend ANC contacts, substantial gaps in content these contacts remain. Therefore, women are not fully benefiting from these services.

Even in relation to other LMIC contexts, our findings suggest that utilization of ANC in rural Bangladesh is strikingly low. Only approximately a quarter of our study participants attended four or more ANC contacts during pregnancy which is comparable with the rural estimate reported in the most recent demographic health survey of Bangladesh [22, 34]. This appears to be lower than the trends in observed other LMICs, where at minimum two-fifths of women receive such care during pregnancy [8, 12, 16]. However, this comparison should be read with caution, as many of these studies are based on the Demographic and Health Survey (DHS) data, which takes into account all ANC contacts irrespective of types of provider while reporting this indicator [35]. We considered contacts with formal health care providers while presenting the coverage of ANC in our study and a negligible proportion of the women reported only attending ANC contacts with informal and untrained providers. Hence, the estimates related to ANC contacts presented in our study can be considered valid and reasonably comparable with other global estimates.

In addition to the low coverage of ANC contacts, the timing of these contacts was poor among our study population. Early initiation of ANC, that is, attending their first ANC contact within the first 12 weeks of pregnancy, is particularly important [3]. Early initiation of ANC provides the opportunity to address pre-existing conditions at the optimal time and is the most effective moment to administer recommended tests and screening and provide counselling [36, 37]. In a systematic review of DHS data of 132 countries, Moller et al. found that approximately a quarter of women in LMICs attend their first ANC contact during the first trimester [38]. The percentage of women receiving their first ANC contact within the first trimester and attending at least four ANC contacts during their pregnancy in our sample was dismal compared estimates of other countries presented in that study [38]. This is also well below other studies which have looked at the same indicator [12]. Among our study population, we found early initiation of ANC to be associated with improved coverage of ANC contacts throughout pregnancy. Among those initiating ANC in the first trimester, the probability of attending at least one ANC contact in each trimester and attending four or more ANC contacts throughout the pregnancy was encouragingly high. This confirms the importance of promoting contact between women and the formal healthcare system at the early stage of their pregnancies. This can be an important lesson for prioritization and programming for promoting MNH and its associated continuum of care.

In our study, we found that education was significantly associated with ANC coverage (i.e. attending any ANC and at least four ANC contacts). Further analysis revealed that the odds of attending four ANC contacts was higher among the higher educated group than that of lower education group (S1 Table). This is consistent with other studies which have found an association between increased coverage of ANC with higher education and wealth status [39, 40]. We also found significant relationships between higher education (10+ years) of men and wealth with ANC coverage, across most categories. We did not detect a significant association between age and ANC coverage, in contrast to another study of 13 west African countries which found an association between early initiation of ANC and women attending at least four ANC contacts with age [17]. We also found a significant relationship between religion and ANC coverage across all indicators, with non-Muslim respondents attending earlier and more ANC contacts compared to Muslim participants. This may reflect a religious stigma within the Muslim community against seeking maternity health services outside of the household, and particularly from male health service providers. This has also been demonstrated in other studies within Bangladesh [41] as well as beyond [42]. Further qualitative studies should explore in more detail factors related to belief systems and individual preferences affecting decisions to seek and use formal health services.

These findings also indicate that when women do access ANC services, there are important gaps in the content of these contacts. Measuring blood pressure was the most commonly performed component in the ANC contacts among our study population. Measuring weight was also commonly performed, as was conducting an abdominal examination. Screening tests were less commonly performed, with three-quarters of women having a urine test, and just over half having a blood screening test performed. This is consistent with the most recent DHS estimate for rural Bangladesh and other studies, which have found measuring blood pressure and weight to be the most commonly performed components of ANC, with screening tests (blood and urine) performed less frequently [8, 12, 17, 22]. This may be at least partially attributable to the system wide weakness of health systems in Bangladesh which is accentuated by the inappropriate level of readiness of health facilities in rural Bangladesh as reported by the Bangladesh Health Facility Survey [30]. However, we also acknowledge that this argument should be placed with caution since our study was done in a small scale and may not representative of the whole country. Some studies have found health facilities to be generally inadequately prepared to deliver ANC according to recommended guidelines [16, 43]. The latest Bangladesh Health Facility Survey of 2014 also highlighted the critical gaps in service availability and readiness of both public and private facilities in regard to MNH services, particularly ANC [30]. Our study was not designed to assess the readiness of health facilities, however it is promising to note that, while not necessarily complete, performance of different recommended components of ANC contacts in our study seems to be among the upper levels of what has been found in other LMICs [15, 17, 39, 44, 45]. Therefore, while women in Netrokona appear to be using ANC services at the lower rate compared to what is observed globally when they access these services, the contacts appear to be relatively more complete than in other settings.

Several factors were significantly associated with the components of ANC performed during contacts. Across all ANC content-indicators, components were more likely to be performed when the ANC contact occurred in the health facility compared to home-based ANC contacts. Results were mixed as to the ANC provider. Interestingly, doctors were less likely to perform physical examinations and counselling but were more likely to conduct screening tests. Similarly, when considering all facility-based health service providers and community-based health workers were more likely to conduct physical examination and counselling, and less likely to conduct screening tests. This suggests that efforts aiming to improve the quality of ANC in rural Bangladesh should take these factors into account when designing approaches. Interestingly, we also found the presence of the woman’s husband during the ANC contact to be associated with administration of screening tests, ultrasound, and abdominal examination. Different studies have reported that involvement or presence of the husband/male partner is associated with the use of skilled MNH services [46–50]. Our findings provide further insights into the potential of improving the content of ANC contacts by promoting male involvement in MNH. Although it requires further exploration and validation, promoting male involvement during ANC contacts may be considered as a strategy for MNH programs focusing on improving MNH services.

Regarding counselling-related aspects, counselling on danger signs was revealed as one of the most neglected components of ANC contacts as per our study findings. Fewer than three-quarters of women attending ANC among our study population were counselled on danger signs during pregnancy. This is generally consistent with other studies which have also found counselling on danger signs to be among the most neglected components [8, 12, 51]. This is an important finding, as ANC contacts provide an optimal platform for educating women on danger signs and how to respond appropriately during obstetric emergencies. Moreover, according to the respondents of our study, the environment is not always optimal for counselling with privacy not systematically maintained. Privacy in maternity care settings has been found to be lacking in a variety of settings [52–55]. This is important not only because privacy and confidentiality are defined as women’s rights and an essential component of respectful maternity care [56], but also because it is necessary for a conducive counselling environment [3, 57]. Women are less likely to be able to express themselves openly and be able to work effectively with a health service provider when they are concerned that their conversations may be overheard. Although we could not look at all the components of ANC counselling in our paper, the poor status of danger sign counselling, care seeking counselling and privacy during ANC contacts can serve as tracer indicators for the policy makers to concentrate on this critical issue and take appropriate strategies to improve the quality of ANC contacts.

Our results indicate that the private sector plays an important role in the provision of ANC services. This is consistent with Campbell et al., who found that private sector has a major market share in providing MNH services in LMICs, despite this sector being generally less monitored, controlled and understood than the public sector [58]. We found that some of the indicators related to the content of ANC contacts were better in the private sector, especially in terms of performing physical examinations, ultrasound and danger sign counselling. This is similar to one of the studies conducted in Brazil, which reported that ANC services delivered in the private sector to be more comprehensive [40]. Future research should examine this in more detail, particularly as the most recent maternal mortality survey in Bangladesh (BMMS 2016) indicates that the private sector is increasing in importance in the delivery of MNH services [18].

Overall, our findings suggest that both ANC coverage and content in rural Bangladesh are sub-optimum. Based on this, we recommend future research to understand more fully the factors which prevent women from seeking ANC, both early in pregnancy and the recommended times between conception and birth. It is critical to understand these factors so that the national programs can take course corrective measures to address them appropriately. In tandem, it is also critical for the country to put efforts in place to ensure the content and quality of ANC contacts are improved substantially; so that women can gain the greatest possible benefits when accessing services. All components of ANC should be prioritized, from physical examinations and screening through counselling, which is often neglected. Health services providers should be trained to effectively counsel women and families on MNH issues and promote a positive counselling environment.

## Limitations

We want to acknowledge some of the limitations of our study and present some of our attempts to address them adequately. Firstly, the results presented in this study are from a cross-sectional survey. Therefore, we cannot infer causality between the explanatory variables and outcomes of interest related to ANC. We have interpreted the results with caution and presented the associations after adjustments where necessary. However, we did not have enough information (eg. availability of health services, distance, family composition, birth order, etc.) to build a comprehensive model based on any conceptual framework (eg. Andersen’s model) for presenting the factors that affect ANC coverage and content of ANC contacts. The factors presented in this paper are exploratory and should be explored in details through future studies adopting a behavioural model of health care utilization [59]. The potential of recall bias is another important limitation of this study. Based on the pregnancy outcomes, the women’s recall regarding ANC contacts and content may have changed. We adopted our questionnaire from validated national and international survey tools and finalized it after pre-testing to minimize this bias. The other limitation of the study could be ‘recall error’ as we accepted up to 12 months of recall. Even if in the recent past, it is possible that women misremembered the events of the individual ANC contacts, or that they did not necessarily understand the different components. However, we feel that the data collectors were well trained and had the capacity to clarify different elements of the ANC contacts and content of ANC contacts when necessary. Moreover, the recall period in our study was 12 months, which is much shorter than the 3 to 5 years recall period that is accepted by other surveys generating national estimates [18, 22, 35]. Another potential limitation could be the social desirability bias. We recruited data collectors from local communities who are familiar with the local culture, language and norms.

The content of ANC contacts presented in the paper are based on the WHO’s recent ANC recommendation and Bangladesh Maternal Health Strategy [3, 24]. However, it is not comprehensive of all the contents recommended as we were limited by the availability of data. In addition, while we were able to assess the different components of ANC, which were carried out during ANC contacts, we were not able to assess the quality of the services provided. We also did not have any qualitative data to explain some of the preference and practices related to ANC contacts and content of care. This is particularly important for counselling. We were limited by the availability of data on danger sign and care seeking counselling during ANC contacts. We could not report on other aspects of ANC counselling like post-partum family planning, newborn are preparedness, birth preparedness, etc. Counselling for MNH care is not likely to be effective if a top-down information-dissemination approach is being used [57]. It is more likely to be effective and result in behavior change when health services providers demonstrate positive interpersonal and counselling skills [60], which we could not address in our study. Future research should investigate the quality of ANC contacts, particularly the counselling conducted so that women are able to make and act on plans to improve their health and that of their children.

## Conclusion

Ensuring adequate coverage of ANC and maintaining adherence to the recommended ANC guidelines has the potential to save the lives of women and newborns globally and contribute towards achieving the ambitious targets encompassed by the SDGs. Our study demonstrates that coverage of ANC remains poor in Netrokona, Bangladesh, both in terms of timing of initiation of ANC and in the number of contacts. Moreover, glaring gaps remain in the content of the care received which women access these services. Therefore, it is recommended that overcoming barriers to health services access be prioritized and well as efforts to ensure the content and the quality of ANC, so that women and newborns throughout Bangladesh are able to fully benefit from these services and realize their rights to health.

## Supporting information

S1 Table Associations between coverage of ANC contacts and background characteristics, among women with a recent history of childbirth (N = 737). (DOCX)

S2 Table Relationships between status of receiving content of ANC and background characteristics, among women who have attended ANC (N = 454). (DOCX)

S1 Data Dataset. (DTA)
