# Correction: Antenatal care in rural Bangladesh: Gaps in adequate coverage and content

**DOI:** 10.1371/journal.pone.0238551

**Published:** 2020-08-27

**Authors:** Abu Bakkar Siddique, Janet Perkins, Tapas Mazumder, Mohammad Rifat Haider, Goutom Banik, Tazeen Tahsina, Md. Jahurul Islam, Shams El Arifeen, Ahmed Ehsanur Rahman

The following information is missing from this article’s Funding statement:

This study also received financial support from Enfants du Monde, Geneva, Switzerland, by its own funds and through funds received from Geneva Federation for Cooperation (“Fédération genevoise de coopération”).

In addition, the following is missing from the Acknowledgments:

We thank Enfants du Monde for providing financial support for this study, as described in the Funding statement.
