# Supplementary material for: Antenatal care in rural Bangladesh: Gaps in adequate coverage and content
Source: PLoS One. 2018 Nov 19;13(11):e0205149. doi: 10.1371/journal.pone.0205149 (PMC6242304; doi:10.1371/journal.pone.0205149)
Supplement: S1 Table — (DOCX) [file pone.0205149.s001.docx]

**S1 Table: Associations between coverage of ANC contacts and background characteristics, among women with a recent history of childbirth (N=737)**

| Background characteristic | Any ANC | | Early initiation of ANC | | ANC in  all 3 trimesters | | ≥4 ANC | | Early initiation of ANC +  ≥4 ANC | | ANC in  all 3 trimesters  + ≥4 ANC | |
| --- | --- | --- | --- | --- | --- | --- | --- | --- | --- | --- | --- | --- |
|  | OR (CI) | AOR (CI) | OR (CI) | AOR (CI) | OR (CI) | AOR (CI) | OR (CI) | AOR (CI) | OR (CI) | AOR (CI) | OR (CI) | AOR (CI) |
| **Age of woman** |  |  |  |  |  |  |  |  |  |  |  |  |
| 15-24 | **ref** | **ref** | **ref** | **ref** | **ref** | **ref** | **ref** | **ref** | **ref** | **ref** | **ref** | **ref** |
| 25-34 | 0.7 (0.5,0.9) | 0.8 (0.5,1.1) | 1.1 (0.6,1.7) | 1.0 (0.6,1.6) | 1.0 (0.6,1.8) | 0.9 (0.5,1.7) | 0.7 (0.5,1.1) | 0.7 (0.5,1.1) | 0.9 (0.5,1.7) | 0.8 (0.4,1.5) | 1.0 (0.6,1.8) | 0.9 (0.5,1.6) |
| 35+ | 0.6 (0.3,1.1) | 0.7 (0.4,1.2) | 0.6 (0.2,1.6) | 0.3 (0.1,1.1) | 0.6 (0.2,1.9) | 0.3 (0.1,1.3) | 0.9 (0.5,1.7) | 1.0  (0.5,1.8) | 0.4 (0.1,1.6) | 0.2 (0.0,1.3) | 0.4 (0.1,1.7) | 0.2 (0.0,1.3) |
| **Education- women** |  |  |  |  |  |  |  |  |  |  |  |  |
| 0-4 years | **ref** |  | **ref** | **ref** | **ref** | **ref** | **ref** | **ref** | **ref** | **ref** | **ref** | **ref** |
| 5-9 years | 1.2 (0.9,1.7) | 1.0 (0.7,1.4) | 0.7 (0.4,1.2) | 0.5 (0.3,0.9) | 0.9 (0.5,1.7) | 0.8 (0.4,1.5) | 1.1 (0.7,1.5) | 1.0 (0.7,1.5) | 1.2 (0.6,2.4) | 0.8 (0.4,1.8) | 1.1 (0.6,2.2) | 0.8 (0.4,1.7) |
| ≥10 years | 10.0 (3.5,28.6) | 4.9 (1.5,16.1) | 2.4 (1.2,4.6) | 1.1 (0.4,2.9) | 3.8 (1.9,7.7 | 2.1  (0.7,5.9) | 6.0 (3.4,10.7) | 3.3 (1.5,7.0) | 7.1 (3.4,14.9) | 2. 1(0.7,6.3) | 7.1 (3.4,14.9) | 2.5 (0.8,7.7) |
| **Education-husband** |  |  |  |  |  |  |  |  |  |  |  |  |
| 0-4 years | **ref** |  | **ref** | **ref** | **Ref** | **ref** | **ref** | **ref** | **ref** | **ref** | **ref** | **ref** |
| 5-9 years | 1.4 (1.1,2.0) | 1.2 (0.8,1.7) | 1.1 (0.6,1.9) | 0.9 (0.5,1.7) | 1.1 (0.6,2.0) | 0.8  (0.4,1.6) | 1.2 (0.8,1.8) | 1.0 (0.7,1.5) | 1.5 (0.7,2.9) | 1.1 (0.5,2.3) | 1.5  (0.7,2.9) | 1.1  (0.5,2.3) |
| ≥10 years | 3.6  (1.8,7.1) | 1.3 (0.6,3.0) | 3.0 (1.6,5.7) | 1.7 (0.7,4.4) | 3.6 (1.8,7.2) | 1.4 (0.5,3.8) | 4.1  (2.4,6.9) | 1.7 (0.9,3.5) | 7.2 (3.6,14.7) | 2.7 (1.0,7.6) | 6.6 (3.2,13.6) | 2.3 (0.8,6.5) |
| **Religion** |  |  |  |  |  |  |  |  |  |  |  |  |
| Muslim | **ref** |  | **ref** | **ref** | **Ref** | **ref** | **ref** | **ref** | **ref** | **ref** | **ref** | **ref** |
| Other | 1.4 (0.8,2.2) | 1.1 (0.7,1.9) | 3.4 (2.0,6.1) | 3.4 (1.8,6.2) | 4.2 (2.3,7.7) | 3.8 (2.0,7.3) | 2.0 (1.3,3.3) | 1.7 (1.1,2.8) | 4.8 (2.5,8.9) | 3.9 (1.9,7.6) | 4.9 (2.6,9.2) | 4.0 (2.0,8.0) |
| **Wealth Quintile** |  |  |  |  |  |  |  |  |  |  |  |  |
| Lowest | **ref** |  | **ref** | **ref** | **Ref** | **ref** | **ref** | **ref** | **ref** | **ref** | **ref** | **ref** |
| Second | 1.7  (1.1,2.7) | 1.6 (1.0,2.5) | 1.3 (0.5,3.2) | 1.5 (0.6,3.8) | 1.1 (0.4,3.1) | 1.1 (0.4,3.3) | 0.8 (0.5,1.4) | 0.8 (0.4,1.3) | 1.0 (0.3,3.0) | 0.8 (0.3,2.6) | 1.0 (0.3,3.0) | 0.8 (0.3,2.6) |
| Middle | 1.1 (0.7,1.8) | 1.0 (0.6,1.6) | 1.7 (0.7,4.1) | 1.6 (0.6,4.0) | 2.0 (0.7,5.2) | 1.7 (0.6,4.7) | 0.7 (0.4,1.3) | 0.6 (0.4,1.1) | 1.3 (0.4,3.6) | 0.9 (0.3,2.7) | 1.5 (0.5,4.1) | 0.9 (0.3,2.7) |
| Fourth | 1.5 (0.9,2.3) | 1.3 (0.8,2.1) | 2.2 (1.0,5.3) | 2.4 (1.0,6.0) | 1.7 (0.6,4.6) | 1.6 (0.5,4.5) | 0.9 (0.5,1.5) | 0.8 (0.4,1.4) | 1.5 (0.5,4.1) | 1.0 (0.3,3.1) | 1.5 (0.5,4.1) | 1.0 (0.3,3.1) |
| Highest | 3.9 (2.3,6.6) | 2.5 (1.4,4.5) | 2.3 (1.1,5.2) | 1.7 (0.6,4.5) | 2.7 (1.1,6.6) | 1.5 (0.5,4.6) | 1.9 (1.2,3.2) | 1.1  (0.6,2.0) | 3.6 (1.5,9.0) | 1.4 (0.5,4.1) | 3.4 (1.4,8.9) | 1.2 (0.4,3.8) |
