# Supplementary material for: Antenatal care in rural Bangladesh: Gaps in adequate coverage and content
Source: PLoS One. 2018 Nov 19;13(11):e0205149. doi: 10.1371/journal.pone.0205149 (PMC6242304; doi:10.1371/journal.pone.0205149)
Supplement: S2 Table — (DOCX) [file pone.0205149.s002.docx]

**S2 Table: Relationships between status of receiving content of ANC and background characteristics, among women who have attended ANC (N=454)**

| Background characteristic | Physical examination (blood pressure& abdominal examination& weight measured) | | | Screening tests  (urine test& blood test& USG) | | | Counselling (DSC & CSC & privacy) | | | All | | |
| --- | --- | --- | --- | --- | --- | --- | --- | --- | --- | --- | --- | --- |
|  | % | OR (CI) | AOR (CI) | % | OR (CI) | AOR (CI) | % | OR (CI) | AOR (CI) | % | OR (CI) | AOR (CI) |
| **Age of woman** |  |  |  |  |  |  |  |  |  |  |  |  |
| 15-24 | 74.1 | **ref** | **ref** | 42.9 | **ref** | **ref** | 43.6 | **ref** | **ref** | 29.1 | **ref** | **ref** |
| 25-34 | 81.1 | 1.6 (1.0,2.4) | 1.5 (0.9,2.4) | 61.2 | 2.1 (1.4,3.1) | 2.3 (1.5,3.4) | 47.4 | 1.2 (0.8,1.7) | 1.1 (0.7,1.7) | 43.0 | 1.8 (1.2,2.7) | 1.9 (1.2,2.9) |
| 35+ | 65.3 | 0.7 (0.3,1.4) | 0.7 (0.3,1.4) | 43.0 | 1.0 (0.5,2.0) | 1.0 (0.5,2.2) | 39.5 | 0.8 (0.4,1.7) | 0.8 (0.4,1.7) | 38.8 | 1.5 (0.8,3.1) | 1.6 (0.7,3.4) |
| **Education- women** |  |  |  |  |  |  |  |  |  |  |  |  |
| 0-4 years | 74.8 | **ref** | **ref** | 42.5 | **ref** | **ref** | 42.4 | **ref** | **ref** | 30.5 | **ref** | **ref** |
| 5-9 years | 75.9 | 1.1 (0.7,1.7) | 0.9 (0.5,1.4) | 54.2 | 1.6 (1.1,2.3) | 1.3 (0.8,2.0) | 43.4 | 1.0 (0.7,1.5) | 0.8 (0.5,1.3) | 36.1 | 1.3 (0.9,1.9) | 1.1 (0.7,1.7) |
| ≥10 years | 86.8 | 2.2 (1.0,5.1) | 0.8 (0.3,2.2) | 76.2 | 4.3 (2.2,8.5) | 2.2 (0.9,5.2) | 61.5 | 2.2 (1.2,4.0) | 0.8 (0.4,1.9) | 61.0 | 3.6 (1.9,6.6) | 1.5 (0.6,3.4) |
| **Education-husband** |  |  |  |  |  |  |  |  |  |  |  |  |
| 0-4 years | 70.8 | **ref** | **ref** | 43.9 | **ref** | **ref** | 40.0 | **ref** | **ref** | 30.3 | **ref** | **ref** |
| 5-9 years | 80.8 | 1.7 (1.1,2.8) | 1.6 (0.9,2.7) | 54.7 | 1.5 (1.0,2.3) | 1.0 (0.6,1.6) | 47.0 | 1.3 (0.9,2.0) | 1 (0.7,1.6) | 36.3 | 1.3 (0.9,2.0) | 0.9 (0.5,1.4) |
| ≥10 years | 90.8 | 4.1 (1.6,10.3) | 3.0 (1.0,9.1) | 76.6 | 4.2 (2.2,8.1) | 1.6 (0.7,3.6) | 64.0 | 2.7 (1.5,4.8) | 1.6 (0.7,3.6) | 64.9 | 4.3 (2.3,7.8) | 1.8 (0.8,4.0) |
| **Religion** |  |  |  |  |  |  |  |  |  |  |  |  |
| Other | 84.3 | 1.7 (0.8,3.6) | 1.4 (0.6,3.0) | 74.2 | 3.1 (1.7,5.6) | 2.6 (1.3,5.1) | 54.2 | 1.5 (0.9,2.6) | 1.2 (0.7,2.2) | 58.3 | 2.8 (1.6,4.8) | 2.1 (1.2,3.9) |
| Muslim | 75.6 | **ref** | **ref** | 48.4 | **ref** | **ref** | 43.8 | **ref** | **ref** | 33.5 | **ref** | **ref** |
| **Wealth Quintile** |  |  |  |  |  |  |  |  |  |  |  |  |
| Lowest | 79.3 | **ref** | **ref** | 32.2 | **ref** | **ref** | 36.7 | **ref** | **ref** | 23.4 | **ref** | **ref** |
| Second | 67.9 | 0.6 (0.3,1.1) | 0.6 (0.3,1.2) | 44.5 | 1.7 (0.9,3.1) | 1.7  (0.9,3.2) | 29.6 | 0.7 (0.4,1.4) | 0.8 (0.4,1.5) | 23.1 | 1.0 (0.5,2.0) | 1.0 (0.5,2.1) |
| Middle | 70.3 | 0.6 (0.3,1.3) | 0.6 (0.3,1.2) | 51.9 | 2.3 (1.2,4.3) | 1.9 (1.0,3.7) | 48.8 | 1.6 (0.9,3.1) | 1.7 (0.9,3.4) | 37.0 | 1.9 (1.0,3.8) | 1.7 (0.8,3.4) |
| Fourth | 74.5 | 0.8 (0.4,1.6) | 0.7 (0.3,1.5) | 53.0 | 2.4 (1.3,4.4) | 2.2 (1.1,4.3) |  | 1.7 (0.9,3.1) | 1.7 (0.9,3.4) | 38.3 | 2.0 (1.0,4.0) | 2.0 (1.0,4.1) |
| Highest | 88.7 | 2.0 (0.9,4.5) | 1.4 (0.6,3.5) | 68.8 | 4.6 (2.5,8.5) | 2.9 (1.4,6.0) | 57.8 | 2.4 (1.3,4.2) | 2.3 (1.2,4.7) | 54.6 | 3.9 (2.1,7.4) | 2.9 (1.4,6.0) |
| **Total** | 76.8 | - | - | 51.6 | - | - | 45.2 | - | - | 36.6 | - | - |
